# Binding of the Antagonist Caffeine to the Human Adenosine Receptor hA_2A_R in Nearly Physiological Conditions

**DOI:** 10.1371/journal.pone.0126833

**Published:** 2015-05-20

**Authors:** Ruyin Cao, Giulia Rossetti, Andreas Bauer, Paolo CarIoni

**Affiliations:** 1 German Research School for Simulation Sciences (joint venture of RWTH Aachen University and Forschungszentrum Jülich GmbH), D-52425, Jülich, Germany; 2 Computational Biomedicine, Institute for Advanced Simulation (IAS-5), Forschungszentrum Jülich GmbH, D-52425, Jülich, Germany; 3 Institute of Neuroscience and Medicine (INM-9), Forschungszentrum Jülich GmbH, D-52425, Jülich, Germany; 4 Jülich Supercomputing Centre (JSC), Forschungszentrum Jülich GmbH, D-52425, Jülich, Germany; 5 Institute of Neuroscience and Medicine (INM-2), Forschungszentrum Jülich GmbH, D-52425, Jülich, Germany; University of Michigan, UNITED STATES

## Abstract

Lipid composition may significantly affect membrane proteins function, yet its impact on the protein structural determinants is not well understood. Here we present a comparative molecular dynamics (MD) study of the human adenosine receptor type 2A (hA_2A_R) in complex with caffeine—a system of high neuro-pharmacological relevance—within different membrane types. These are POPC, mixed POPC/POPE and cholesterol-rich membranes. 0.8-μs MD simulations unambiguously show that the helical folding of the amphipathic helix 8 depends on membrane contents. Most importantly, the distinct cholesterol binding into the cleft between helix 1 and 2 stabilizes a specific caffeine-binding pose against others visited during the simulation. Hence, cholesterol presence (~33%-50% in synaptic membrane in central nervous system), often neglected in X-ray determination of membrane proteins, affects the population of the ligand binding poses. We conclude that including a correct description of neuronal membranes may be very important for computer-aided design of ligands targeting hA_2A_R and possibly other GPCRs.

## Introduction

Increasingly emerging experiments point to an important role of membrane lipid composition in structure/function relationships of G-protein coupled receptors (GPCRs)—the largest membrane protein family in mammals [1–4]. For instance, in rhodopsin, adding (1-palmitoyl-2-oleoylphosphatidylethanolamine) POPE lipids into POPC (1-palmitoyl-2-oleoylphosphatidylcholine) lipid bilayers affect the equilibrium between two sub-states of the rhodopsin functional cycle [5,6]. Another example is given by the class A GPCR serotonin1A receptor: its binding to the agonist 8-OH-DPAT decreases with cholesterol concentration [7]. This issue is crucial for pharmacological applications, as more than one quarter of FDA-approved drugs target GPCRs [8].

Molecular Dynamics (MD) simulations are being instrumental to assess the effect of native membrane environment on conformational properties of GPCRs. Indeed, MD studies of class A GPCR, beta-2 adrenergic receptor, using four different membrane types with or without cholesterol, have suggested that the stability of the receptor “ionic lock” [9] varies with different lipid compositions [4]. In addition, 1.6 μs MD simulations of rhodopsin embedded in (1-stearoyl-2-docosahexaenoyl-phosphatidylcholine) SDPC/SDPE (1-stearoyl-2 docosahexaenoyl-phosphatidylethanolamine) lipid bilayer, in the presence of cholesterol, have lead to the conclusion that specific cholesterol-rhodopsin binding modulates the TM1-TM2-TM7 helices/helix 8 interactions, essential for the receptor’s activation [10]. Finally, MD simulations indicates that the stabilization of the amphipathic helix 8 (H8) of the class C GPCR mGluR2 receptor increases with cholesterol concentration and that such stabilization depends also on membrane thickness [3].

Here we use MD simulations to elucidate the effect of membrane composition on an antagonist-bound neuronal GPCR. This is the class A GPCR human adenosine receptor type 2A (hA_2A_R) [11]—highly localized in the so-called “striatum” of the brain [12]—in complex with the antagonist caffeine (CFF, chemical formula in S1 Fig). CFF binding to hA_2A_R may lead to neuroprotection [13–17]. It prevents apoptotic cell death in a Parkinson’s' model [18]. The effect on passing from artificial lipid bilayers to conditions near to real neuronal membranes, where cholesterol content varies from 33% to 50% [19], is investigated by performing 0.8 μs-long MD simulations on three systems, named in sequence as **I**, **II** and **III** (see Table 1). **I** is composed by pure POPC lipid bilayer (see S1 Fig), which is commonly used for MD studies on adenosine receptor [20–25]. **II** is a bilayer of equally mixed POPC and POPE lipids (S1 Fig). **III** resembles the synaptic membrane (42% POPC, 34% POPE and 25% of cholesterol molecules, S1 Fig), where hA_2A_R is expressed [26] and the binding to CFF exerts its beneficial neuroprotection effects [16]. Notably, **III** markedly differs from the artificial membrane mimics (detergent n-nonyl-β-D-glucopyranoside), where the CFF/hA_2A_R complex is embedded for X-ray structure determination [27].

**Table 1:** Composition of the three systems simulated here.

System	Lipid ratio	#POPC molecules	#POPE molecules	#Cholesterol molecules	#Water molecules	Ions	#Total atoms
**I**	POPC	495	-	-	26839	152 Na+; 165 Cl-	152,414
**II**	POPC/POPE (0.50:0.50)	247	249	-	27244	152 Na+; 165 Cl-	151,522
**III**	POPC/POPE /Cholesterol (0.42:0.34:0.24)	248	204	141	25703	148 Na+; 161 Cl-	151,834

## Results and Discussion

### Convergence Analysis

The degree of convergence of the 800 ns long MD simulations of systems **I**, **II** and **III** is here investigated by using the so-called ‘all-to-all RMSD analysis’ [28]. This assembles pairs of Cα atoms' RMSDs in matrices along an MD simulation. In the last 400 ns, the matrices of the three systems (S2 Fig) show a "leave-and-return pattern", a converged-alike feature according to [28], see supporting information. On longer time-scales, this feature is not observed. This suggests that the protein conformations of the three systems might have reached a fair convergence in the last 400 ns timescale here. Consistently with this fact, the Cα RMSDs oscillate around average values in such time scale (Panel A in S3 Fig). The properties presented here are therefore calculated for the last 400 ns.

### Overall Fold

**Fig 1:**
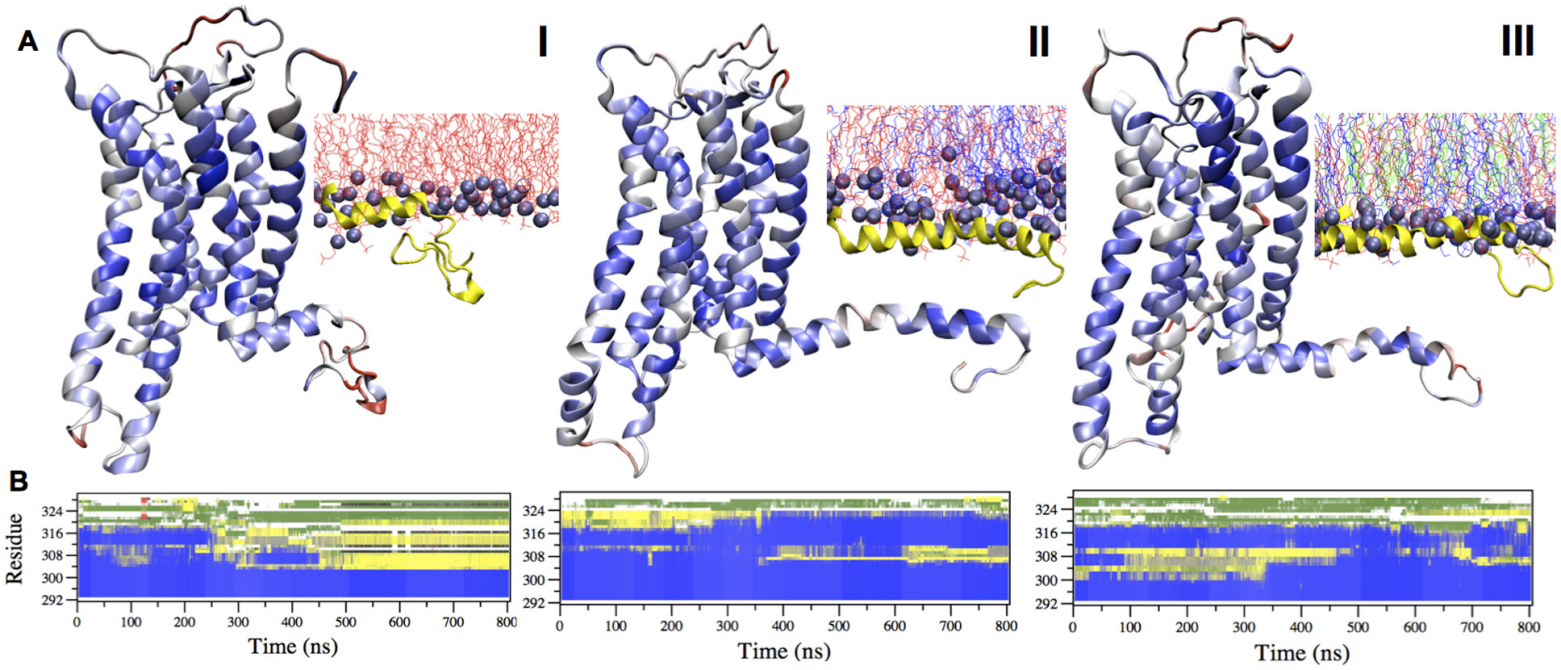
Membrane-sensitive folding of H8. **A)** The cartoon representations of receptor’s backbone of systems **I**–**III** are shown in blue to red according to residues’ increased flexibility, as emerging from the so-called PAD index values [32]; the inserted panel shows the location of H8 (in yellow cartoon) in each membrane; POPC, POPE and cholesterol molecules are shown in red, blue, green lines respectively. The phosphorous atoms are shown as violet Van de Waals spheres. **B)** Secondary structure content of H8-including C segment (res 292 to 329) (^292^REFRQTFRKIIRSHVLRQQEPFKAAAAHHHHHHHHHHH^329^) is reported as a function of the simulated time. β sheet, α helix, coil and bend, and turn are shown in red, blue, white, green and yellow, respectively.

The typical seven transmembrane helices are maintained across the three systems during the overall trajectory (Panel B in S3 Fig). However, the fold of H8, located at the membrane-cytoplasm interface, which was not solved in the X-ray structure [27], shows membrane-sensitive conformations. Indeed residues 292–317 of H8 preserve a helical conformation in **II** and **III** only (Fig 1). Yet, the helical content of H8 decreases in **I**: residues 305–317 unfolded into flexible loop after 250 ns MD (Fig 1). This is associated with two features, which are present only in **I**: a very large increase of the Cα RMSD (Panel A in S3 Fig), and the presence of two non-overlapping blocks in the matrix calculated with the all-to-all RMSD analysis (S2 Fig). The latter is a signature of a significant, irreversible transition between two distinct conformations [28]. The different stability of H8 across the three systems is likely to be related to an increase of membrane thickness on passing from **I** to **II**, and, more, to **III**, observed here (see Section ‘**Membrane Structure**’ below for details). As results, H8 in **II** and **III** is only half-exposed to the solvent, being the other half immersed in the membrane. This stabilizes H8, because this helix is amphipathic (Panel B in S4 Fig). Such stabilization is not present for system **I**, where H8 is more solvent-exposed because of the thinner thickness of the bilayer. H8 is a key structural element for hA_2A_R function, as it connects the transmembrane helices interacting with ligands with the cytoplasmic C terminus coupling with alpha-actinin (type 2), dopamine receptors (types 2 and 3), glutamate mGlu5 receptors and other regulatory GPCRs [29,30]. Hence, our simulations point to the importance of using proper membrane environment to study this neurotransmitter receptor. Our findings share similarities with an NMR study of the structurally-related class A GPCR human β_2_ adrenergic receptor, where H8 is helical in DMSO and disordered in water [31]. Also for the class C GPCR mGluR2 receptor [3], mixed POPC/cholesterol membrane was shown to stabilizes the helical structure of the H8, whereas the pure POPC membrane induces a disruption of H8.

The flexibility of the three systems, here analyzed in terms of the so-called PAD index for backbone atoms [32], is similar for the three systems, with the exceptions of the N-term of helix H1 and the second extracellular loop ECL2 (S5 Fig), which are significantly more flexible in **I**.

### Binding Site

**Fig 2:**
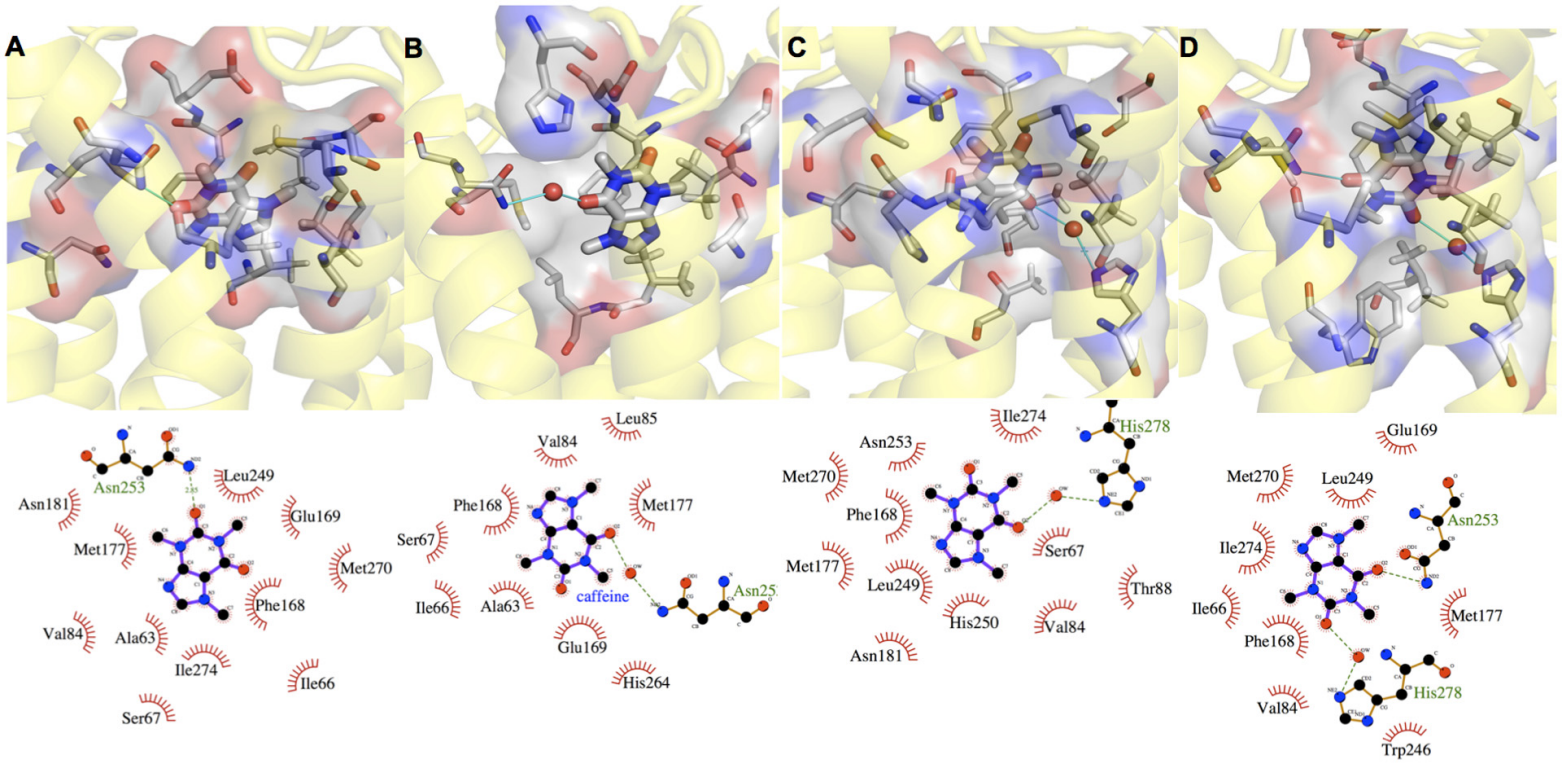
CFF’s most populated binding poses in systems I-III (A-D). For each binding pose, the upper panel shows the protein backbone in yellow cartoon, CFF and residues interacting with CFF in thick and thin sticks, respectively. Water molecules forming H-bonds with CFF and residues are represented as red sphere; the lower panel shows the corresponding 2-d chart.

**Fig 3:**
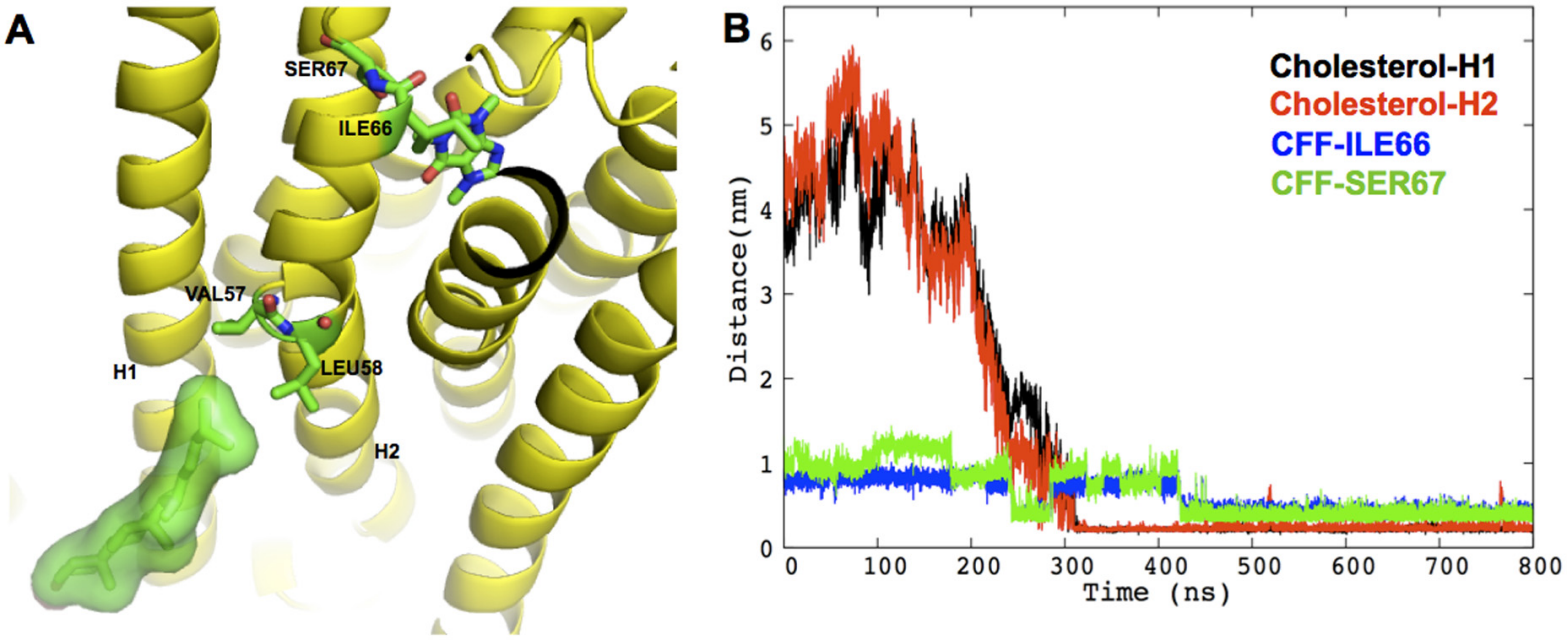
Specific cholesterol binding to hA_2A_R A) Cartoon showing cholesterol-binding pose in H1/H2 cleft in system III. The receptor is shown in yellow cartoon; the cholesterol molecule is shown as green sticks surrounded by its solvent accessible surface; CFF, cholesterol-interacting residues, VAL57, LEU58, as well as CFF-interacting residues ILE66, SER67 are shown as green sticks with oxygen and nitrogen atoms colored in red and blue, respectively. **B) The diffusion of cholesterol into of the H1/H2 cleft enhances hydrophobic contacts between CFF and H2.** The minimum distances between the specific cholesterol molecule and H1 (residues 5–34), between cholesterol and H2 (residues 41–67), between C5@CFF and heavy atoms of ILE66 and SER67 side chains, are shown in black, red, blue and green, respectively.

CFF exhibits multiple binding poses (A-D in Fig 2) in receptor binding cavity across the three systems, comparable to what found for adenosine in this receptor [33]. Most of the identified binding poses are similar to those found in the 0.069-μs MD study of a H8-truncated CFF/hA_2A_R complex [34]. Our poses yet differ from those in the 0.005-μs MD study [35], possibly because of the large difference (more than two orders of magnitude) between our time scale and theirs. The population of the CFF poses depends on the type of membrane environment. A (38%), B (29%), and C (94%) are the most populated poses for **I**, **II**, **III**, respectively (see Fig 2 and Table 2). Notably, the pose in the X-ray structure (D) [27] (Fig 2), is not the most populated one in any of the three systems (see S1 Text). C is almost the only pose assumed by the antagonist in the physiologically relevant system **III**. It is stabilized by hydrophobic contacts between the CFF/C5 methyl group and ILE66 and SER67 side chains on the extracellular side of H2 (panel A in Fig 3). This stabilizing interaction may be triggered by the diffusion of a cholesterol molecule, already after 0.3 μs, to the cleft between H1 and H2 (panel B in Fig 3). Indeed, this specific cholesterol binding induces conformational rearrangements of VAL57, LEU58, ILE66 and SER67 (S6 Fig), which in turn result in the enhanced stabilization of the hydrophobic interaction between CFF and H2 residues. We conclude that the cholesterol very likely drives specific pose for CFF. The calculated lateral diffusion coefficient of cholesterol molecules around the receptor is not too dissimilar from that of cholesterol molecules in the proximity of the lipids (5*10^−8^ cm^2^ s^-1^, 8*10^−8^ cm^2^ s^-1^ respectively, S2 Text and S7 Fig), suggesting that the observed cholesterol binding event is not strongly dependent on cholesterol’s starting location. Notably, in the absence of cholesterol molecules (systems **I** and **II**), one POPC molecule replaces cholesterol in the binding cleft. Hence, this receptor’s binding cleft seems to act like an anti-diffusion trap for lipids and, more, for cholesterol molecules, showing higher specificity for the latter. Interestingly, in the μs-MD simulations of apo hA_2A_R, Lyman et al. also detected the specific cholesterol presence between helices H1 and H2 [23].

**Table 2:** Populations of CFF binding poses (%) detected across systems I-III over the last 400 ns of MD simulated time.

BP Index	I	II	III
A	38.1		-
B	-	29.1	-
C	31.9	5.8	92.4
D	-	-	3.8

**Fig 4:**
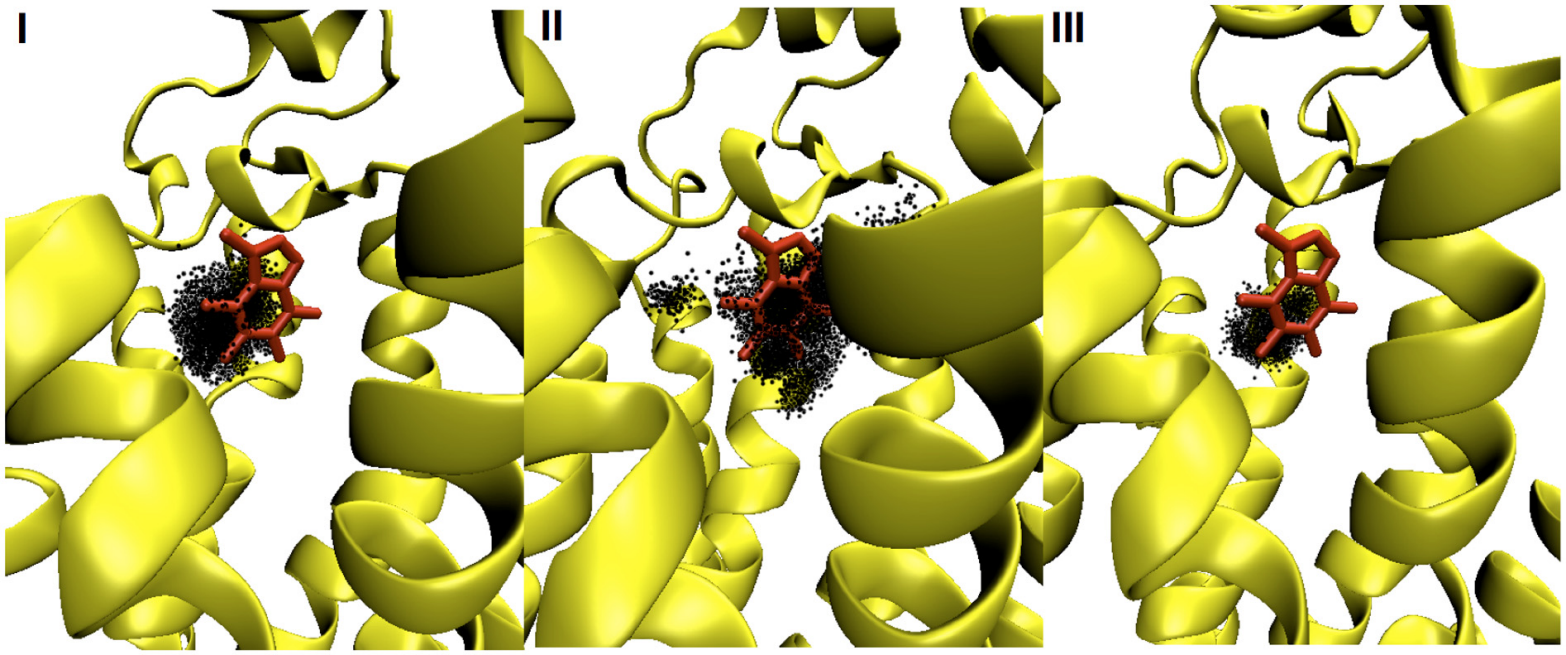
The distribution of CFF center of mass within the ligand-binding cavity of hA_2A_R across systems I-III. The receptor and CFF are shown as yellow cartoon and red sticks, respectively. CFF center of mass at each collected frame over the last 400 ns of MD simulated time is depicted as one black dot.

Next, we investigated the mobility of CFF, i.e. the different extent of roto-translation of the ligand inside the cavity across the three systems over the last 400 ns. This mobility is measured in terms of the orientational flipping angle (defined in the method section, see S8 Fig) and the CFF center of mass (Fig 4), sampled along the simulation time. Not surprisingly, **III** features the smallest fluctuations of both quantities, as the ligand is mostly in the C conformation. **II** exhibits the largest fluctuations whilst **I** features intermediate values (see Fig 4 and S8 Fig).

The MD-averaged number of water molecules in the binding cavity of **II** (32 molecules) is much larger than those of **I** and, more, of **III** (21 and 16 molecules, respectively, see S9 Fig). Hence, this study corroborates the plausible hypothesis that enhanced hydration of binding cavity increases ligand dynamics in **II** [33].

### Membrane Structure

The primary amine group of POPE (in **II** and **III**) forms intra and intermolecular hydrogen bonding with the lipids' phosphate groups (S2 Table) [36]. Water- and receptor-lipid hydrogen bonds are comparable across the three systems (S2 Table).

The different membrane composition affects its thickness. The latter increases on passing from **I** to **II**, and from **II** to **III**. The latter observation is consistent with the experimental observation that the presence of cholesterol causes an increase of thickness of lipid bilayers [37]. These features are shown by a plot of phosphate groups’ density distributions (panel A in S4 Fig). The area per lipid decreases from 0.61 nm^2^ (system **I**) to 0.56 nm^2^ (system **II**) and 0.50 nm^2^ (system **III**), see S2 Table. This is consistent with the fact that the average area per lipid of pure POPC is greater than that of pure POPE membrane [38]. Hence, the area per lipid is anti-correlated with the thickness of the membrane, consistently with what already found in ref. [39].

The POPC and headgroups’ dipole moments turn out to be oriented differently on passing from **I** to the other two systems. Let us define the PN vector (from the phosphorus atom to the nitrogen atom of one lipid headgroup, see S10 Fig) and the angle (Φ_PN_) between the PN vector and the axis perpendicular to the lipid bilayer surface (z-axis). The POPC headgroups’ dipoles turn out to be perpendicular to z-axis in system **I**, with a large standard deviation (Φ_PN_ ~ 90(36) degrees, see S11 Fig). Instead, in systems **II** and **III**, these headgroups show a bivariate distribution with two shallow peaks around Φ_PN_ ~ 65 degree and Φ_PN_ ~ 115. Interestingly, the POPE headgroups are oriented perpendicular to the z-axis (Φ_PN_ ~ 90(28) degrees and 90(29) for systems **II** and **III**, respectively).

In system **III**, the POPC/POPE ratio is 1.2:1. This differs from that of system **II**, which features a POPC/POPE ratio of 1:1. To test whether this difference in POPC/POPE ratio plays a role for protein structural, we performed an additional simulation where we replaced 24 POPE molecules of system **II** with 24 POPC molecules. The results are rather similar to the ones of system **II** and are reported in SI (S12 Fig).

## Conclusion

Both hA_2A_R fold and CFF binding dynamics are sensitive to the lipid environment where hA_2A_R is embedded. The lipid polar headgroups also exhibit varied dipole orientations in different membrane environments. Most importantly, the presence of cholesterol in the membrane is shown to drastically affect CFF binding pose population and mobility. X-ray studies commonly crystallize ligand/receptor complexes in detergent mimics [40], without the physiologically high concentration of cholesterol. The artificial environment is found here to affect the population of ligand poses drastically: the pose found in the X-ray structure, at 3.6-Å of resolution, is the most populated one in none of our 0.8 μs-long MD simulations. This study suggests that computer-aided studies of hA_2A_R in nearly physiological conditions may give key contributions to the investigation of receptor's function as well as to the development of CFF derivatives that retain CFF's neuroprotective benefits with much higher affinity for the target than CFF [41,42].

## Materials and Methods

### Homology Modeling of hA_2A_R

hA_2A_R is a class A GPCR, composed of 7 transmembrane helices (H1–H7) and a helix lying at the membrane-cytoplasm interface (H8). The X-ray structure of the CFF/hA_2A_R complex has been solved at a 3.6-Å resolution (PDBid: 3RFM) [27]. Amino acid sequence after residue 317 was deleted to remove the highly mobile cytoplasmic C terminus. The truncated sequence was joined by a polyhistidine tag (residues 318–329). Residues ^1^MPIMGS^6^, ^150^KEGKNHSQ^157^, ^306^HVLRQQEPFKAAAAHHHHHHHHHH^329^ are not detected in the crystal structure. Moreover the crystalized receptor contains 8 mutations (A54L, T88A, R107A, K122A, L202A, L235A, V239A, S277A). The missing regions were complemented and the mutations were mutated back to wild type by multiple-template-based homology modeling (S1 Table).

12 X-ray structures of hA_2A_R are deposited in the Protein Data Bank [15,27,43–47]. Among these we selected 3RFM and 4 other templates with resolution below 3.0 Å (3VG9, 3EML, 4EIY, 2YDV). Notice that the 2YDV template where hA_2A_R is bound with an agonist N-ETHYL-5'-CARBOXAMIDO (NEC), is the only one used for modeling the residues 291–325 in the C terminus without including any other residues, as it has the longest resolved helix H8 among all the available hA_2A_R X-ray structures. 100 models using the 5 templates were generated in Modeller 9.11 [48]. The best model, in terms of both DOPE score [49] and stereochemistry PROCHECK analysis [50] underwent to loop refinement procedure [48]. 500 models were generated. The best model was selected as the optimal initial model for MD simulations.

The first six residues in the N terminus are predicted as a helical segment. The missing 8 residues (^150^KEGKNHSQ^157^) in the loop connecting helix 4 and helix 5 are also so as to form a short helical segment. As for the missing residues at the C terminus, residues ^306^HVLRQQEPFKAAAAHHHH^323^ are modeled as the H8 [46]. The last six residues, all histidines, are modeled as a loop. The backbone Root Mean Square Deviation (RMSD) between the model and 3RFM is 0.9 Å. The backbone RMSD of the residues located in hA_2A_R ligand binding site (within 7.0 Å of CFF) against their counterparts in 3RFM is 3.3 Å.

### Simulation Details

Membrane models in systems **I**, **II** and **III** were generated using the MemBuilder tool [51]. The inflateGRO code [52] was used to pack lipids around the hA_2A_R constructed in the previous step. The systems were inserted in a simulation box of size 14.8 nm x 11.1 nm x 10.0 nm. With this choice, the minimum distance between periodic images of the protein was larger than 1.5 nm in all systems. Water, sodium and chloride ions were added in order to solvate and neutralize the systems at an ionic strength of 0.15 M. The final systems comprised ~150,000 atoms (Table 1).

The AMBER99SB-ILDN force fields [53], the Slipids [54,55], the TIP3P [56] force fields were used for the protein and ions, the lipids, and the water molecules respectively. The General Amber force field (GAFF) parameters [57] were used for CFF, along with the RESP atomic charge using Gaussian 09 [58] with the HF-6-31G* basis set [59,60]. MD simulations were performed using Gromacs v4.5.5 package [61] on JUROPA supercomputer. The Particle Mesh Ewald method [62] was used to treat the long-range electrostatic interaction with a real space cutoff of 1.2 nm. A 1.2 nm cutoff was used for the short-range non-bonded interaction. A timestep of 2 fs was set. The LINCS algorithm [63] was applied to constrain all bonds involving hydrogen atoms. Constant temperature and pressure conditions were achieved via independently coupling protein, lipids, solvent and ions to Nosè-Hoover thermostat [64] at 310 K and Andersen-Parrinello-Rahman Barostat [65] at 1 atm. For each system, the receptor in the free state underwent minimization, 1-ns simulated annealing and 10-ns equilibration with positional restraint using a force constant of 1000 kJ mol^-1^ nm^-2^ on the heavy atoms of the protein. 40-ns equilibration was further carried out with positional restraint on the side chains of the residues within the binding cavity (residues within 7.0 Å of CFF on backbone alignment to 3RFM [27]). This allowed water molecules to diffuse into the ligand-binding cavity. Next, CFF was inserted so as to fit the conformation it has in the X-ray structure [27] using backbone alignment in Pymol [66]. Energy minimization, annealing, 20-ns equilibration with positional restraint on the side chains of the residues belonging to the binding cavity and the CFF were performed before removing all the restraints. Then 0.8 μs MD at 310 K and 1 atm was performed for **I**, **II**, **III**, with one frame collected every 20 ps. The starting binding pose of CFF resembled fairly that in the X-ray counterpart (RMSD = 1.4 Å, 0.8 Å, 2.3 Å for **I**, **II**, **III**).

### Trajectory Analysis

The RMSD, pairwise RMSD matrices [28] and secondary structure content are calculated over the entire trajectory with g_rms, do_dssp of the Gromacs v4.6.5 package [61]. The CFF orientational flipping angle is defined as arccos(μ_τ_·μ_ι_), where μ_ι_ is the vector in the plane of the bicyclic core of CFF, chosen so that it faces toward the extracellular side in the initial frame ι of the trajectory; μ_τ_ is the vector at each frame τ of the trajectory. Also this quantity is calculated over the entire trajectory.

The following properties are calculated over the last 400 ns of the three MD simulations: (i) The PAD flexibility index, using a in house code [67,68]. (ii) The density profile of lipid phosphate groups along the z axis, using g_density in Gromacs v4.6.5 package [61]. (iii) The CFF binding poses, identified using the Gromos cluster algorithm [69] with a 2-Å RMSD cutoff on alignment of protein backbone. The g_cluster module of Gromacs v4.6.5 [61] has been used. (iv) CFF center of mass is defined by the vector r_i_ of the coordinates of CFF center of mass at a frame i, upon protein backbone alignment. (v) The average number of water molecules in the ligand binding cavity of hA_2A_R is calculated by following the similar procedure in [33,70]. Namely, for each system, the number of water molecules in a rectangular box, centered in the binding site and incorporating residues A63, T88, F168, M177, W246, L249, H250, N253, I274, H278 (i.e. residue within 4.5 Å from CFF in 3RFM [27]), is averaged over the trajectory’s frames. (vi) The average hydrogen bond occupancies are calculated as average number of hydrogen bonds formed between the receptor and lipids ($\rho_{HB}^{prt-lip}$), within lipids ($\rho_{HB}^{lip-lip}$), and between lipids and solvent molecules ($\rho_{HB}^{lip-sol}$), divided by the total number of acceptor oxygen atoms. (vii) The average area per lipid (APL) is calculated with the GridMat-MD program [71]. (viii) The PN vector of one lipid molecule is defined as the vector from the phosphorous atom to the nitrogen atom of its polar headgroup, as defined in [72]. The Φ_PN_ of one lipid molecule is the angle between its PN vector and the z-axis. This is the axis perpendicular to the lipid bilayer surface. For each system, Φ_PN_ of each lipid molecule is sampled over the entire MD simulation for the normalized Φ_PN_ distributions of POPC and POPE lipids. (ix) the lateral diffusion coefficients of cholesterol molecules in **III** are calculated using the Einstein relation [73] for a 60 ns simulation of **III** in the NVT ensemble (see S2 Text for details). Cholesterol molecules are classified as molecules in close proximity of the protein if they have atoms within 0.35 nm of the receptor during the dynamics. The other molecules are classified as 'free'.

Molecular graphics are drawn using Pymol [66], VMD [74] and Ligplot+ [75].

## Supporting Information

S1 Fig Chemical structures of CFF, POPC, POPE and cholesterol molecules. (PDF)

S2 Fig Pairwise RMSD matrix of Cα atoms. (PDF)

S3 Fig Proteins' backbone. (PDF)

S4 Fig Selected properties of systems I-III. (PDF)

S5 Fig Flexibility of individual residues of hA_2A_R. (PDF)

S6 Fig Cholesterol-induced conformational transitions of H2 residues in system III. (PDF)

S7 Fig Lateral Mean-square displacements (MSDs) of two groups of cholesterol molecules in system III. (PDF)

S8 Fig CFF orientational flipping angle. (PDF)

S9 Fig The hydration of the ligand binding cavity of hA_2A_R. (PDF)

S10 Fig Orientation of POPC and POPE headgroups. (PDF)

S11 Fig Distribution of Φ_PN_ of lipid headgroups for systems I-III. (PDF)

S12 Fig Changing the ratio between the lipids in system II. (PDF)

S1 Table Available X-ray structures of hA_2A_R. (PDF)

S2 Table Selected membrane properties of systems I-III. (PDF)

S1 Text Description of most populated CFF binding poses A-D. (PDF)

S2 Text Lateral diffusion coefficient of cholesterol molecules. (PDF)

## References

[1] Niu S-L, Mitchell DC, Litman BJ (2002) Manipulation of cholesterol levels in rod disk membranes by methyl-β-cyclodextrin effects on receptor activation. Journal of Biological Chemistry 277: 20139–20145. 11889130 10.1074/jbc.M200594200

[2] Stone W, Farnsworth C, Dratz E (1979) A reinvestigation of the fatty acid content of bovine, rat and frog retinal rod outer segments. Experimental eye research 28: 387–397. 446567 10.1016/0014-4835(79)90114-3

[3] Bruno A, Costantino G, de Fabritiis G, Pastor M, Selent J (2012) Membrane-sensitive conformational states of helix 8 in the metabotropic Glu2 receptor, a class C GPCR. PloS one 7: e42023 10.1371/journal.pone.0042023 22870276 PMC3411606

[4] Mahmood MI, Liu X, Neya S, Hoshino T (2013) Influence of lipid composition on the structural stability of G-protein coupled receptor. Chemical and Pharmaceutical Bulletin 61: 426–437. 23546002 10.1248/cpb.c12-01059

[5] Soubias O, Teague WE, Hines KG, Mitchell DC, Gawrisch K (2010) Contribution of membrane elastic energy to rhodopsin function. Biophysical Journal 99: 817–824. 10.1016/j.bpj.2010.04.068 20682259 PMC2913204

[6] Soubias O, Gawrisch K (2012) The role of the lipid matrix for structure and function of the GPCR rhodopsin. Biochimica et Biophysica Acta (BBA)-Biomembranes 1818: 234–240. 10.1016/j.bbamem.2011.08.034 21924236 PMC3253906

[7] Pucadyil TJ, Chattopadhyay A (2004) Cholesterol modulates ligand binding and G-protein coupling to serotonin 1A receptors from bovine hippocampus. Biochimica et Biophysica Acta (BBA)-Biomembranes 1663: 188–200. 15157621 10.1016/j.bbamem.2004.03.010

[8] Overington JP, Al-Lazikani B, Hopkins AL (2006) How many drug targets are there? Nature reviews Drug discovery 5: 993–996. 17139284 10.1038/nrd2199

[9] Ballesteros JA, Jensen AD, Liapakis G, Rasmussen SG, Shi L, Gether U, et al (2001) Activation of the β2-adrenergic receptor involves disruption of an ionic lock between the cytoplasmic ends of transmembrane segments 3 and 6. Journal of Biological Chemistry 276: 29171–29177. 11375997 10.1074/jbc.M103747200

[10] Khelashvili G, Grossfield A, Feller SE, Pitman MC, Weinstein H (2009) Structural and dynamic effects of cholesterol at preferred sites of interaction with rhodopsin identified from microsecond length molecular dynamics simulations. Proteins: Structure, Function, and Bioinformatics 76: 403–417. 10.1002/prot.22355 19173312 PMC4101808

[11] Jacobson KA, Ukena D, Padgett W, Daly JW, Kirk KL (1987) Xanthine functionalized congeners as potent ligands at A2-adenosine receptors. Journal of Medicinal Chemistry 30: 211–214. 3806597 10.1021/jm00384a037 PMC3433718

[12] Fink JS, Weaver DR, Rivkees SA, Peterfreund RA, Pollack AE, Adler EM, et al (1992) Molecular cloning of the rat A2 adenosine receptor: selective co-expression with D2 dopamine receptors in rat striatum. Molecular Brain Research 14: 186–195. 1279342 10.1016/0169-328x(92)90173-9

[13] Ross G, Abbott RD, Petrovitch H, Morens DM, Grandinetti A, K. T, et al (2000) Association of coffee and caffeine intake with the risk of parkinson diseases. JAMA: The Journal of the American Medical Association 283: 2674–2679. 10819950 10.1001/jama.283.20.2674

[14] Gomes CV, Kaster MP, Tom AR, Agostinho PM, Cunha RA (2011) Adenosine receptors and brain diseases: neuroprotection and neurodegeneration. Biochimica et Biophysica Acta (BBA)—Biomembranes 1808: 1380–1399. 21145878 10.1016/j.bbamem.2010.12.001

[15] Liu W, Chun E, Thompson AA, Chubukov P, Xu F, Katritch V, et al (2012) Structural basis for allosteric regulation of GPCRs by sodium ions. Science 337: 232–236. 10.1126/science.1219218 22798613 PMC3399762

[16] Postuma RB, Lang AE, Munhoz RP, Charland K, Pelletier A, Moscovich M, et al (2012) Caffeine for treatment of Parkinson disease: A randomized controlled trial. Neurology 79: 651–658. 10.1212/WNL.0b013e318263570d 22855866 PMC3414662

[17] Douna H, Bavelaar BM, Pellikaan H (2012) Neuroprotection in Parkinson's disease: a systematic review of the preclinical data. The Open Pharmacology Journal 6: 12–26.

[18] Nakaso K, Ito S, Nakashima K (2008) Caffeine activates the PI3K/Akt pathway and prevents apoptotic cell death in a Parkinson's disease model of SH-SY5Y cells. Neuroscience Letters 432: 146–150. 10.1016/j.neulet.2007.12.034 18201823

[19] Pfrieger FW (2003) Role of cholesterol in synapse formation and function. Biochimica et Biophysica Acta (BBA)-Biomembranes 1610: 271–280. 12648780 10.1016/s0005-2736(03)00024-5

[20] Rodríguez D, Piñeiro An, Gutiérrez-de-Terán H (2011) Molecular dynamics simulations reveal insights into key structural elements of adenosine receptors. Biochemistry 50: 4194–4208. 10.1021/bi200100t 21480628

[21] Pang X, Yang M, Han K (2013) Antagonist binding and induced conformational dynamics of GPCR A2A adenosine receptor. Proteins: Structure, Function, and Bioinformatics 81: 1399–1410. 10.1002/prot.24283 23508898

[22] Ng HW, Laughton CA, Doughty SW (2013) Molecular dynamics simulations of the adenosine A2a receptor: structural stability, sampling, and convergence. Journal of Chemical Information and Modeling 53: 1168–1178. 10.1021/ci300610w 23514445

[23] Lyman E, Higgs C, Kim B, Lupyan D, Shelley JC, Farid R, et al (2009) A role for a specific cholesterol interaction in stabilizing the apo configuration of the human A2A adenosine receptor. Structure 17: 1660–1668. 10.1016/j.str.2009.10.010 20004169 PMC2796422

[24] Li J, Jonsson AL, Beuming T, Shelley JC, Voth GA (2013) Ligand-dependent activation and deactivation of the human adenosine A2A receptor. Journal of the American Chemical Society 135: 8749–8759. 10.1021/ja404391q 23678995 PMC4120839

[25] Lee JY, Lyman E (2012) Predictions for cholesterol interaction sites on the A2A adenosine receptor. Journal of the American Chemical Society 134: 16512–16515. 10.1021/ja307532d 23005256 PMC3652312

[26] Mori A, Shindou T, Ichimura M, Nonaka H, Kase H (1996) The role of adenosine A2A receptors in regulating GABAergic synaptic transmission in striatal medium spiny neurons The Basal Ganglia V: Springer pp. 119–122. 10.1523/JNEUROSCI.16-02-00605.1996 PMC6578641 8551344

[27] Doré Andrew S, Robertson N, Errey James C, Ng I, Hollenstein K, Tehan B, et al (2011) Structure of the adenosine A2A receptor in complex with ZM241385 and the xanthines XAC and caffeine. Structure 19: 1283–1293. 10.1016/j.str.2011.06.014 21885291 PMC3732996

[28] Grossfield A, Zuckerman DM (2009) Quantifying uncertainty and sampling quality in biomolecular simulations. Annual reports in computational chemistry 5: 23–48. 20454547 10.1016/S1574-1400(09)00502-7 PMC2865156

[29] Ciruela F, Albergaria C, Soriano A, Cuffí L, Carbonell L, Sánchez S, et al (2010) Adenosine receptors interacting proteins (ARIPs): behind the biology of adenosine signaling. Biochimica et Biophysica Acta (BBA)-Biomembranes 1798: 9–20. 19883624 10.1016/j.bbamem.2009.10.016

[30] Gsandtner I, Freissmuth M (2006) A tail of two signals: the C terminus of the A2A-adenosine receptor recruits alternative signaling pathways. Molecular Pharmacology 70: 447–449. 16707626 10.1124/mol.106.026757

[31] Katragadda M, Maciejewski M, Yeagle P (2004) Structural studies of the putative helix 8 in the human β2 adrenergic receptor: an NMR study. Biochimica et Biophysica Acta (BBA)-Biomembranes 1663: 74–81. 15157609 10.1016/j.bbamem.2004.01.012

[32] Caliandro R, Rossetti G, Carloni P (2012) Local fluctuations and conformational transitions in proteins. Journal of Chemical Theory and Computation 8: 4775–4785. 26605630 10.1021/ct300610y

[33] Lee JY, Lyman E (2012) Agonist dynamics and conformational selection during microsecond simulations of the A2A adenosine receptor. Biophysical Journal 102: 2114–2120. 10.1016/j.bpj.2012.03.061 22824275 PMC3341534

[34] Sabbadin D, Ciancetta A, Moro S (2014) Bridging molecular docking to membrane molecular dynamics to investigate GPCR—ligand recognition: the human A2A adenosine receptor as a key study. Journal of Chemical Information and Modeling 54: 169–183. 10.1021/ci400532b 24359090

[35] Liu Y, Burger SK, Ayers PW, Vöhringer-Martinez E (2011) Computational study of the binding Modes of caffeine to the adenosine A2A receptor. The Journal of Physical Chemistry B 115: 13880–13890. 10.1021/jp2022049 21970461

[36] McIntosh TJ (1996) Hydration properties of lamellar and non-lamellar phases of phosphatidylcholine and phosphatidylethanolamine. Chemistry and physics of lipids 81: 117–131. 8810046 10.1016/0009-3084(96)02577-7

[37] Hung W-C, Lee M-T, Chen F-Y, Huang HW (2007) The condensing effect of cholesterol in lipid bilayers. Biophysical Journal 92: 3960–3967. 17369407 10.1529/biophysj.106.099234 PMC1868968

[38] Murzyn K, Róg T, Pasenkiewicz-Gierula M (2005) Phosphatidylethanolamine-phosphatidylglycerol bilayer as a model of the inner bacterial membrane. Biophysical Journal 88: 1091–1103. 15556990 10.1529/biophysj.104.048835 PMC1305115

[39] Elmore DE (2006) Molecular dynamics simulation of a phosphatidylglycerol membrane. FEBS letters 580: 144–148. 16359668 10.1016/j.febslet.2005.11.064

[40] Serebryany E, Zhu GA, Yan EC (2012) Artificial membrane-like environments for in vitro studies of purified G-protein coupled receptors. Biochimica et Biophysica Acta (BBA)-Biomembranes 1818: 225–233. 10.1016/j.bbamem.2011.07.047 21851807

[41] Rivera-Oliver M, Díaz-Ríos M (2014) Using caffeine and other adenosine receptor antagonists and agonists as therapeutic tools against neurodegenerative diseases: A review. Life Sciences 101: 1–9. 10.1016/j.lfs.2014.01.083 24530739 PMC4115368

[42] Armentero MT, Pinna A, Ferré S, Lanciego JL, Müller CE, Franco R (2011) Past, present and future of A2A adenosine receptor antagonists in the therapy of Parkinson's disease. Pharmacology & Therapeutics 132: 280–299. 21810444 10.1016/j.pharmthera.2011.07.004 PMC3205226

[43] Congreve M, Andrews SP, Doré AS, Hollenstein K, Hurrell E, Langmead CJ, et al (2012) Discovery of 1,2,4-triazine derivatives as adenosine A2A antagonists using structure based drug design. Journal of Medicinal Chemistry 55: 1898–1903. 10.1021/jm201376w 22220592 PMC3308197

[44] Hino T, Arakawa T, Iwanari H, Yurugi-Kobayashi T, Ikeda-Suno C, Nakada-Nakura Y, et al (2012) G-protein-coupled receptor inactivation by an allosteric inverse-agonist antibody. Nature 482: 237–240. 10.1038/nature10750 22286059 PMC3303121

[45] Jaakola V-P, Griffith MT, Hanson MA, Cherezov V, Chien EYT, Lane JR, et al (2008) The 2.6 Angstrom crystal structure of a human A2A adenosine receptor bound to an antagonist. Science 322: 1211–1217. 10.1126/science.1164772 18832607 PMC2586971

[46] Lebon G, Warne T, Edwards PC, Bennett K, Langmead CJ, Leslie AGW, et al (2011) Agonist-bound adenosine A2A receptor structures reveal common features of GPCR activation. Nature 474: 521–525. 10.1038/nature10136 21593763 PMC3146096

[47] Xu F, Wu H, Katritch V, Han GW, Jacobson KA, Gao Z-G, et al (2011) Structure of an agonist-bound human A2A adenosine receptor. Science 332: 322–327. 10.1126/science.1202793 21393508 PMC3086811

[48] Fiser A, Šali A (2003) Modeller: generation and refinement of homology-based protein structure models. Methods in Enzymology. pp. 461–491. 14696385 10.1016/S0076-6879(03)74020-8

[49] Shen M-y, Sali A (2006) Statistical potential for assessment and prediction of protein structures. Protein Science 15: 2507–2524. 17075131 10.1110/ps.062416606 PMC2242414

[50] Laskowski RA, Macarthur MW, Moss DS, Thornton JM (1993) PROCHECK: a program to check the stereochemical quality of protein structures. Journal of Applied Crystallography 26: 283–291.

[51] Ghahremanpour MM, Arab SS, Aghazadeh SB, Zhang J, van der Spoel D (2014) MemBuilder: a web-based graphical interface to build heterogeneously mixed membrane bilayers for the GROMACS biomolecular simulation program. Bioinformatics 30: 439–441. 10.1093/bioinformatics/btt680 24273238

[52] Kandt C, Ash WL, Peter Tieleman D (2007) Setting up and running molecular dynamics simulations of membrane proteins. Methods 41: 475–488. 17367719 10.1016/j.ymeth.2006.08.006

[53] Best RB, Hummer G (2009) Optimized molecular dynamics force fields applied to the helix—coil transition of polypeptides. The Journal of Physical Chemistry B 113: 9004–9015. 10.1021/jp901540t 19514729 PMC3115786

[54] Jämbeck JPM, Lyubartsev AP (2012) Derivation and systematic validation of a refined all-atom force field for phosphatidylcholine lipids. The Journal of Physical Chemistry B 116: 3164–3179. 10.1021/jp212503e 22352995 PMC3320744

[55] Jämbeck JPM, Lyubartsev AP (2012) An extension and further validation of an all-atomistic force field for biological membranes. Journal of Chemical Theory and Computation 8: 2938–2948. 26592132 10.1021/ct300342n

[56] Jorgensen W, Chandrasekhar J, Madura J, Impey R, Klein M (1983) Comparison of simple potential functions for simulating liquid water. J Chem Phys 79: 926–935.

[57] Wang JM, Wolf RM, Caldwell JW, Kollman PA, Case DA (2004) Development and testing of a general amber force field. Journal of Computational Chemistry 25: 1157–1174. 15116359 10.1002/jcc.20035

[58] Frisch MJ, Trucks GW, Schlegel HB, Scuseria GE, Robb MA, Cheeseman JR, et al (2009) Gaussian 09, Revision A.02. Wallingford CT.

[59] Wang JM, Cieplak P, Kollman PA (2000) How well does a restrained electrostatic potential (RESP) model perform in calculating conformational energies of organic and biological molecules? Journal of Computational Chemistry 21: 1049–1074.

[60] Case DA, Cheatham TE 3rd, Darden T, Gohlke H, Luo R, Merz KM Jr., et al (2005) The Amber biomolecular simulation programs. Journal of Computational Chemistry 26: 1668–1688. 16200636 10.1002/jcc.20290 PMC1989667

[61] Van Der Spoel D, Lindahl E, Hess B, Groenhof G, Mark AE, Berendsen HJC (2005) GROMACS: Fast, flexible, and free. Journal of Computational Chemistry 26: 1701–1718. 16211538 10.1002/jcc.20291

[62] Darden T, York D, Pedersen L (1993) Particle mesh Ewald: An N⋅log(N) method for Ewald sums in large systems. The Journal of Chemical Physics 98: 10089–10092.

[63] Hess B, Bekker H, Berendsen HJC, Fraaije JGEM (1997) LINCS: A linear constraint solver for molecular simulations. Journal of Computational Chemistry 18: 1463–1472.

[64] Hünenberger P (2005) Thermostat Algorithms for Molecular Dynamics Simulations Advanced Computer Simulation: Springer Berlin Heidelberg pp. 105–149.

[65] Parrinello M, Rahman A (1981) Polymorphic transitions in single crystals: A new molecular dynamics method. Journal of Applied Physics 52: 7182–7190.

[66] DeLano WL (2002) The PyMOL molecular graphics system.

[67] Musiani F, Ippoliti E, Micheletti C, Carloni P, Ciurli S (2013) Conformational fluctuations of UreG, an intrinsically disordered enzyme. Biochemistry 52: 2949–2954. 10.1021/bi4001744 23560717

[68] Dibenedetto D, Rossetti G, Caliandro R, Carloni P (2013) A molecular dynamics simulation-based interpretation of nuclear magnetic resonance multidimensional heteronuclear spectra of α-synuclein· dopamine adducts. Biochemistry 52: 6672–6683. 10.1021/bi400367r 23964651

[69] Daura X, Gademann K, Jaun B, Seebach D, van Gunsteren WF, Mark AE (1999) Peptide folding: when simulation meets experiment. Angewandte Chemie International Edition 38: 236–240.

[70] Grossfield A, Pitman MC, Feller SE, Soubias O, Gawrisch K (2008) Internal hydration increases during activation of the G-protein-coupled receptor rhodopsin. Journal of Molecular Biology 381: 478–486. 10.1016/j.jmb.2008.05.036 18585736 PMC3987891

[71] Allen WJ, Lemkul JA, Bevan DR (2009) GridMAT—MD: A grid—based membrane analysis tool for use with molecular dynamics. Journal of computational chemistry 30: 1952–1958. 10.1002/jcc.21172 19090582

[72] Jurkiewicz P, Cwiklik L, Vojtíšková A, Jungwirth P, Hof M (2012) Structure, dynamics, and hydration of POPC/POPS bilayers suspended in NaCl, KCl, and CsCl solutions. Biochimica et Biophysica Acta (BBA)-Biomembranes 1818: 609–616. 10.1016/j.bbamem.2011.11.033 22155683

[73] Allen MP, Tildesley DJ, Banavar JR (2008) Computer simulation of liquids. Physics Today 42: 105–106. 10.1007/s10464-008-9187-7 18597168

[74] Humphrey W, Dalke A, Schulten K (1996) VMD: visual molecular dynamics. Journal of molecular graphics 14: 33–38. 8744570 10.1016/0263-7855(96)00018-5

[75] Laskowski RA, Swindells MB (2011) LigPlot+: multiple ligand—protein interaction diagrams for drug discovery. Journal of Chemical Information and Modeling 51: 2778–2786. 10.1021/ci200227u 21919503

