# Supplementary figures and images for: Binding of the Antagonist Caffeine to the Human Adenosine Receptor hA2AR in Nearly Physiological Conditions
Source: PLoS One. 2015 May 20;10(5):e0126833. doi: 10.1371/journal.pone.0126833 (PMC4439127; doi:10.1371/journal.pone.0126833)

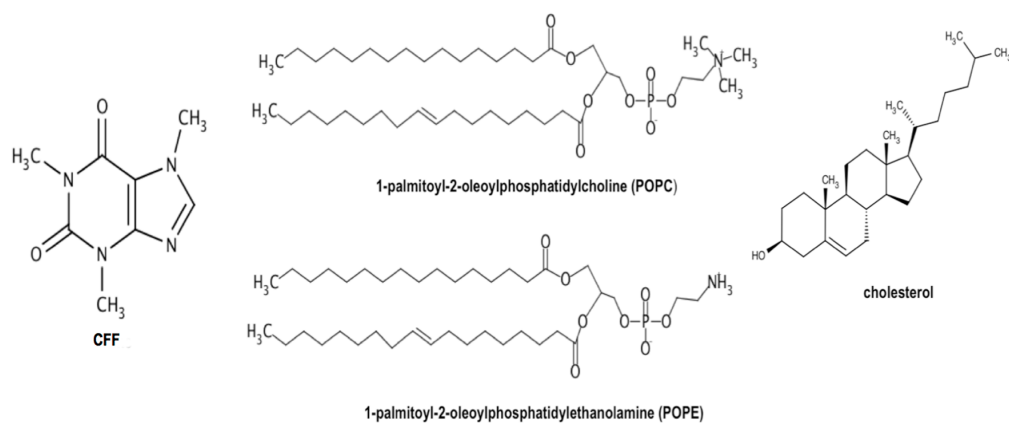

**Supporting Information S1 Fig. Chemical structures of CFF, POPC, POPE and cholesterol molecules.**

Supplement: S1 Fig — (PDF) [file pone.0126833.s001.pdf]
