# Supplementary material for: Binding of the Antagonist Caffeine to the Human Adenosine Receptor hA2AR in Nearly Physiological Conditions
Source: PLoS One. 2015 May 20;10(5):e0126833. doi: 10.1371/journal.pone.0126833 (PMC4439127; doi:10.1371/journal.pone.0126833)
Supplement: S2 Fig — (PDF) [file pone.0126833.s002.pdf]

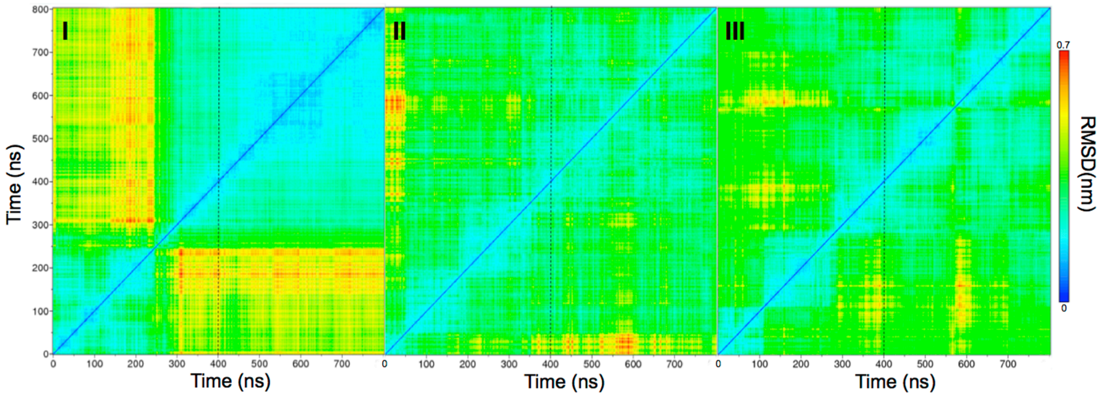

Supporting Information S2 Fig. Pairwise RMSD matrix of Cα atoms. The matrix elements are calculated every 2ns over the 800-ns MD trajectories of systems **I-III** [1]. The color scale shown on the right of the figure quantifies the similarity of the pairs' conformations. The pattern after 400 ns resembles that reported in ref. [1] and it is a signature of convergence [1]. The non-converged and converged substates are separated by black dotted lines.

Supporting References

1. Grossfield A, Zuckerman DM (2009) Quantifying uncertainty and sampling quality in biomolecular simulations. Annual reports in computational chemistry 5: 23-48.
