# Supplementary material for: Binding of the Antagonist Caffeine to the Human Adenosine Receptor hA2AR in Nearly Physiological Conditions
Source: PLoS One. 2015 May 20;10(5):e0126833. doi: 10.1371/journal.pone.0126833 (PMC4439127; doi:10.1371/journal.pone.0126833)
Supplement: S3 Fig — (PDF) [file pone.0126833.s003.pdf]

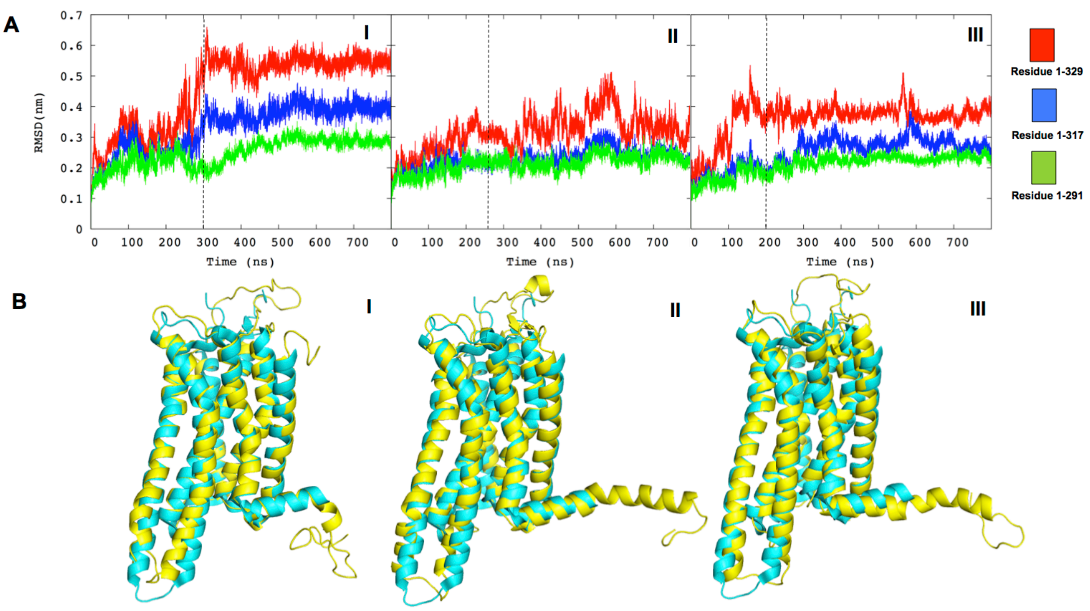

Supporting Information S3 Fig. Proteins' backbone. A) Cα root-mean-square-deviations (RMSDs) of $hA_{2A}R$ across the three systems are plotted as a function of the simulated time. The RMSD values for the full lengths $hA_{2A}R$ (residue 1-329), $hA_{2A}R$ without histidine tag [1] (residue 1-317) and truncated $hA_{2A}R$ (residue 1-291) are represented in red, blue and green, respectively. A dotted black line indicates where RMSDs start to oscillate around averaged values. **B)** Superposition between representative receptor structures obtained by clustering procedure [2] of the three MD simulations (yellow) and the CFF/$hA_{2A}R$ complex X-ray structure [1] (cyan).

Supporting references

1. Doré Andrew S, Robertson N, Errey James C, Ng I, Hollenstein K, Tehan B, et al. (2011) Structure of the adenosine A2A receptor in complex with ZM241385 and the xanthines XAC and caffeine. Structure 19: 1283-1293.

2. Daura X, Gademann K, Jaun B, Seebach D, van Gunsteren WF, Mark AE (1999) Peptide folding: when simulation meets experiment. Angewandte Chemie International Edition 38: 236-240.
