# Supplementary material for: Binding of the Antagonist Caffeine to the Human Adenosine Receptor hA2AR in Nearly Physiological Conditions
Source: PLoS One. 2015 May 20;10(5):e0126833. doi: 10.1371/journal.pone.0126833 (PMC4439127; doi:10.1371/journal.pone.0126833)
Supplement: S4 Fig — (PDF) [file pone.0126833.s004.pdf]

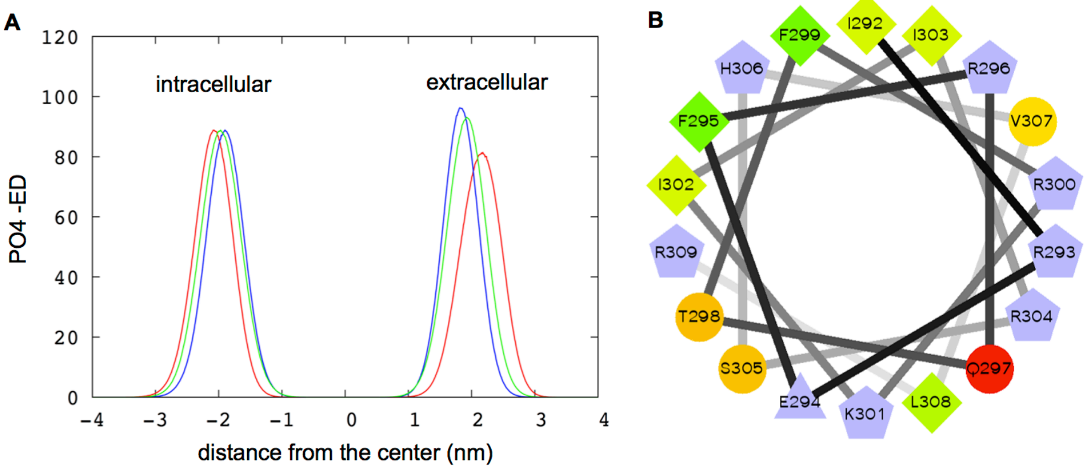

Supporting Information S4 Fig. Selected properties of systems I-III. A) Membrane thickness. MD-averaged distributions of the phosphate groups (PO4-ED) in **I** (blue), **II** (green) and **III** (red), belonging to lipids facing the extracellular (positive distance values) and intracellular (negative distance values), are shown. The peak-to-peak distance is a measure of the lipid bilayer thickness. It is 3.7 nm for **I**, 3.9 nm for **II** and 4.3 nm for **III. B) Properties of helix H8.** Helical wheel diagram of residues ^{292}IREFRQTFRKIIRSHVLR^{309} of H8. These residues are predicted to be helical by the PSIPRED secondary structure prediction server [1]. The diagram is obtained with Helical Wheel Projections server (rzlab.ucr.edu/scripts/wheel/wheel.cgi). The residues are colored as follows: the most hydrophobic residues are green. The amount of green is decreasing proportionally to the hydrophobicity, with zero hydrophobicity coded as yellow. Hydrophilic residues are in red, with pure red being the most hydrophilic (uncharged) residue. The amount of red is decreased proportionally to the hydrophilicity. The charged residues are light blue.

Supporting references

1. McGuffin LJ, Bryson K, Jones DT (2000) The PSIPRED protein structure prediction server. Bioinformatics 16: 404-405.
