# Supplementary material for: Binding of the Antagonist Caffeine to the Human Adenosine Receptor hA2AR in Nearly Physiological Conditions
Source: PLoS One. 2015 May 20;10(5):e0126833. doi: 10.1371/journal.pone.0126833 (PMC4439127; doi:10.1371/journal.pone.0126833)
Supplement: S5 Fig — (PDF) [file pone.0126833.s005.pdf]

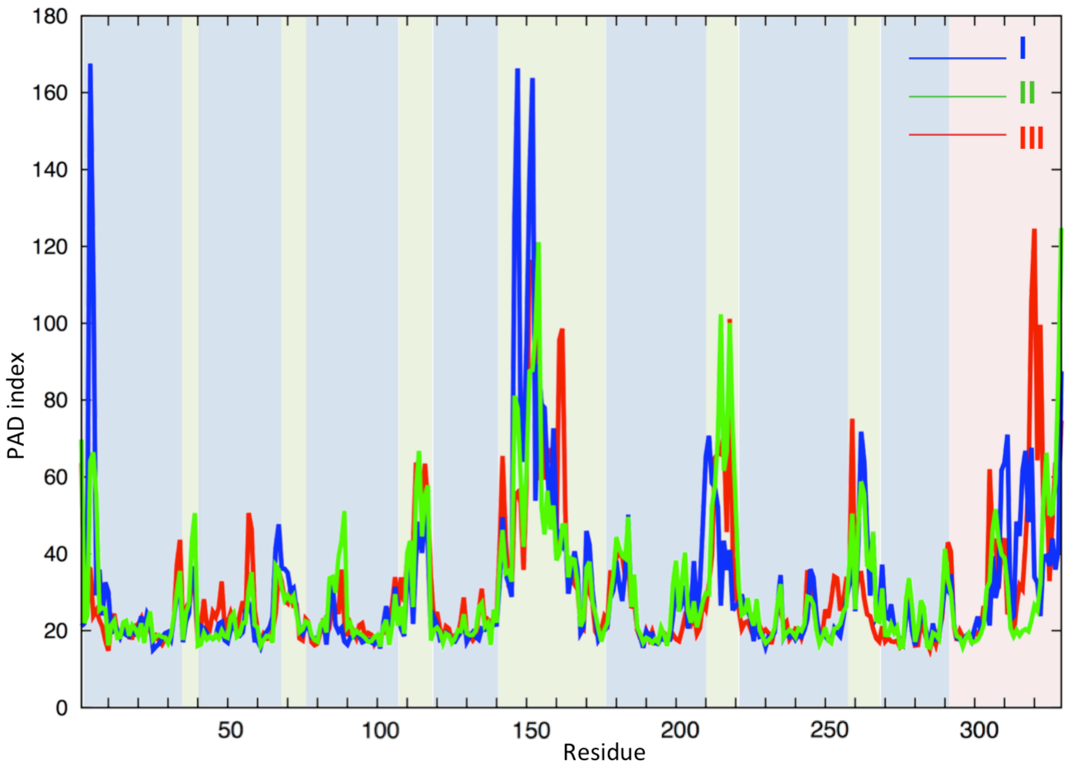

Supporting Information S5 Fig. Flexibility of individual residues of hA_{2A}R. MD-averaged PAD indexes [1] of the receptor in **I** (blue line), **II** (green) and **III** (red). Large PAD values indicate large flexibility for individual residues [1]. The panels in blue, green and red indicate residues belonging to the transmembrane helices H1-H7, to the loops and to the amphipathic helix H8, respectively.

Supporting References

1. Caliandro R, Rossetti G, Carloni P (2012) Local fluctuations and conformational transitions in proteins. Journal of Chemical Theory and Computation 8: 4775-4785.
