# Supplementary material for: Binding of the Antagonist Caffeine to the Human Adenosine Receptor hA2AR in Nearly Physiological Conditions
Source: PLoS One. 2015 May 20;10(5):e0126833. doi: 10.1371/journal.pone.0126833 (PMC4439127; doi:10.1371/journal.pone.0126833)
Supplement: S6 Fig — (PDF) [file pone.0126833.s006.pdf]

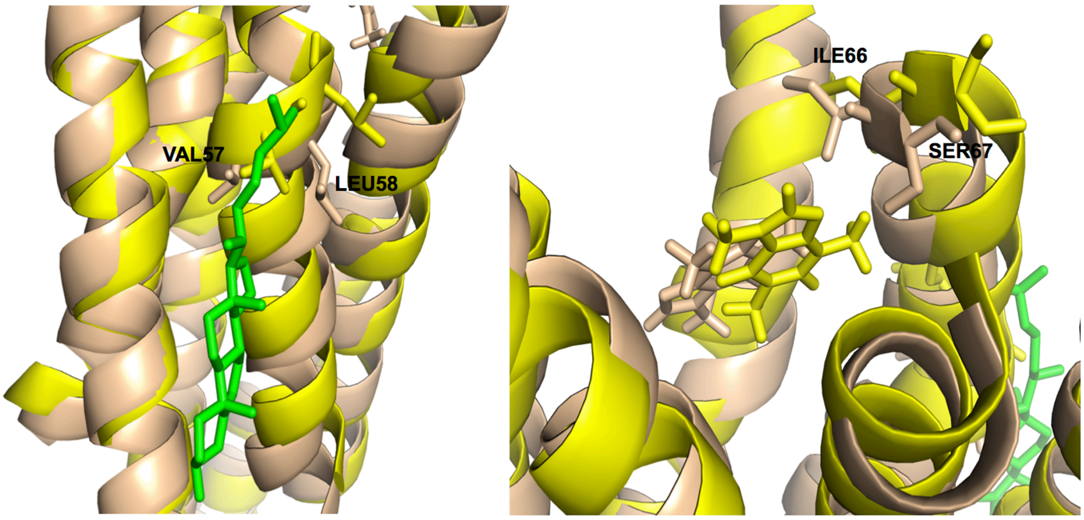

Supporting Information S6 Fig. Cholesterol-induced conformational transitions of H2 residues in system III. Conformational transitions are identified here using the so-called T-PAD analysis, developed by us [1,2]**.** The transitions turn out to involve the residues VAL57, LEU58, ILE66 and SER67. The figure here shows snapshots before (at 150 ns, yellow color) and after the transitions (660 ns, tine color); the cholesterol molecule is shown in green sticks. **Left**: VAL57 and LEU58 rearrange and they interact with the cholesterol molecules. **Right**: ILE66 and SER67 at the binding site change their conformations and interact with CFF.

Supporting References

1. Musiani F, Ippoliti E, Micheletti C, Carloni P, Ciurli S (2013) Conformational fluctuations of UreG, an intrinsically disordered enzyme. Biochemistry 52: 2949-2954.
2. Dibenedetto D, Rossetti G, Caliandro R, Carloni P (2013) A molecular dynamics simulation-based interpretation of nuclear magnetic resonance multidimensional heteronuclear spectra of α-synuclein· dopamine adducts. Biochemistry 52: 6672-6683.
