# Supplementary material for: Binding of the Antagonist Caffeine to the Human Adenosine Receptor hA2AR in Nearly Physiological Conditions
Source: PLoS One. 2015 May 20;10(5):e0126833. doi: 10.1371/journal.pone.0126833 (PMC4439127; doi:10.1371/journal.pone.0126833)
Supplement: S7 Fig — (PDF) [file pone.0126833.s007.pdf]

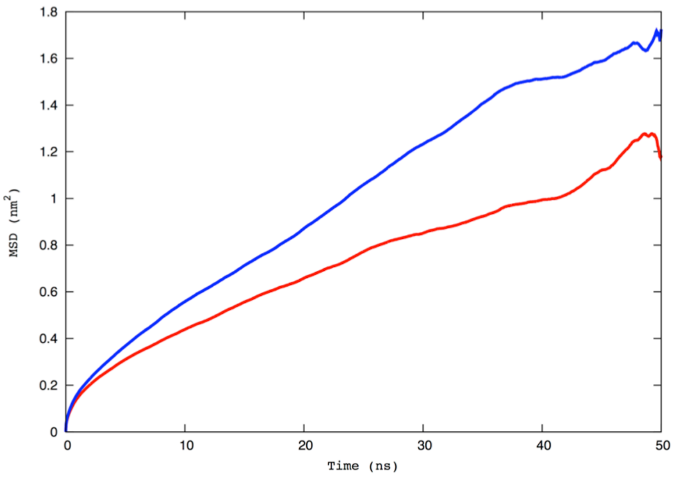

Supporting Information S7 Fig. Lateral Mean-square displacements (MSDs) of two groups of cholesterol molecules in system III. MSDs (mean square displacements) of bound and free cholesterol molecules over the MD simulation in NVT ensemble are shown in red and blue, respectively. The lateral diffusion coefficients of bound cholesterol molecules and free cholesterol molecules are $5*10^{-8}$ cm^2 s^{-1}, $8*10^{-8}$ cm^2 s^{-1}, respectively.
