# Supplementary material for: Binding of the Antagonist Caffeine to the Human Adenosine Receptor hA2AR in Nearly Physiological Conditions
Source: PLoS One. 2015 May 20;10(5):e0126833. doi: 10.1371/journal.pone.0126833 (PMC4439127; doi:10.1371/journal.pone.0126833)
Supplement: S8 Fig — (PDF) [file pone.0126833.s008.pdf]

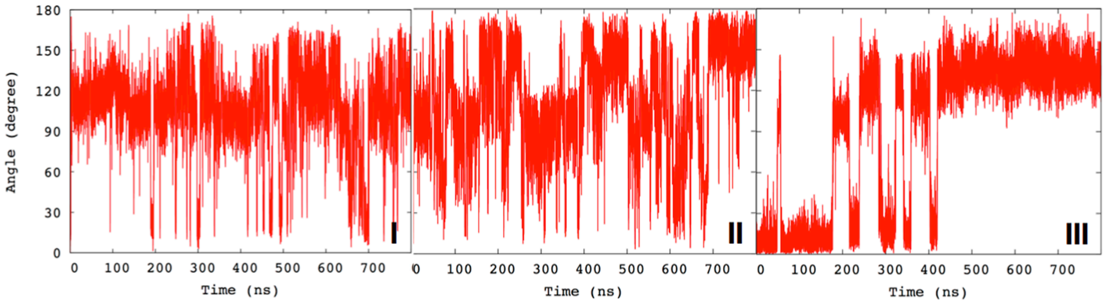

Supporting Information S8 Fig. CFF orientational flipping angle. For each system, **I**-**III**, the CFF orientational flipping angle is plotted as a function of MD simulated time over the entire trajectory.
