# Supplementary material for: Binding of the Antagonist Caffeine to the Human Adenosine Receptor hA2AR in Nearly Physiological Conditions
Source: PLoS One. 2015 May 20;10(5):e0126833. doi: 10.1371/journal.pone.0126833 (PMC4439127; doi:10.1371/journal.pone.0126833)
Supplement: S9 Fig — (PDF) [file pone.0126833.s009.pdf]

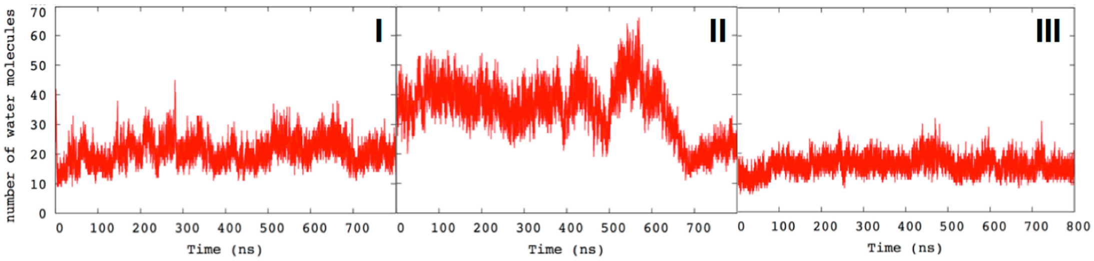

Supporting Information S9 Fig. The hydration of the ligand binding cavity of $hA_{2A}R$. For each system, **I**-**III**, the number of water molecules in the ligand binding cavity of $hA_{2A}R$ is plotted as a function of MD simulated time over the entire trajectory.
