# Supplementary material for: Binding of the Antagonist Caffeine to the Human Adenosine Receptor hA2AR in Nearly Physiological Conditions
Source: PLoS One. 2015 May 20;10(5):e0126833. doi: 10.1371/journal.pone.0126833 (PMC4439127; doi:10.1371/journal.pone.0126833)
Supplement: S10 Fig — (PDF) [file pone.0126833.s010.pdf]

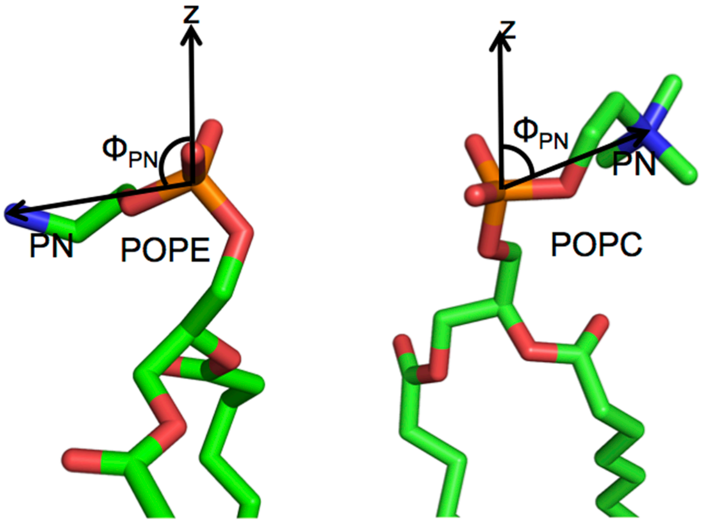

Supporting Information S10 Fig. Orientation of POPC and POPE headgroups. This is defined in terms of the PN vector and the Φ_{PN} the angle. The first points from the P to N atom, the second is the angle between PN and the z axis. The latter is orthogonal to the membrane (see main text for details).
