# Supplementary material for: Binding of the Antagonist Caffeine to the Human Adenosine Receptor hA2AR in Nearly Physiological Conditions
Source: PLoS One. 2015 May 20;10(5):e0126833. doi: 10.1371/journal.pone.0126833 (PMC4439127; doi:10.1371/journal.pone.0126833)
Supplement: S11 Fig — (PDF) [file pone.0126833.s011.pdf]

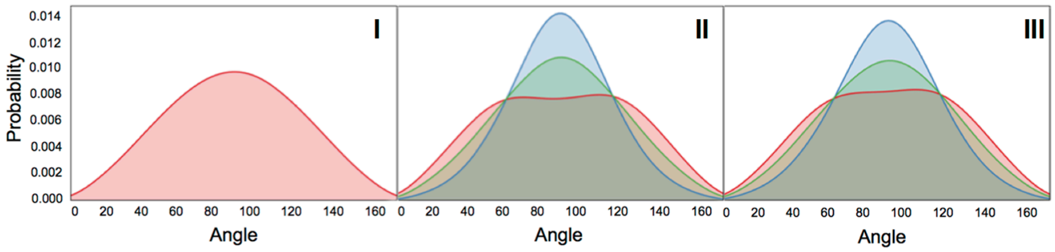

Supporting Information S11 Fig. Distribution of Φ_{PN} of lipid headgroups for systems I-III. Normalized distribution of Φ_{PN}. POPC headgroups, POPE headgroups and overall lipid headgroups are plotted in red, blue and green, respectively.
