# Supplementary material for: Binding of the Antagonist Caffeine to the Human Adenosine Receptor hA2AR in Nearly Physiological Conditions
Source: PLoS One. 2015 May 20;10(5):e0126833. doi: 10.1371/journal.pone.0126833 (PMC4439127; doi:10.1371/journal.pone.0126833)
Supplement: S12 Fig — (PDF) [file pone.0126833.s012.pdf]

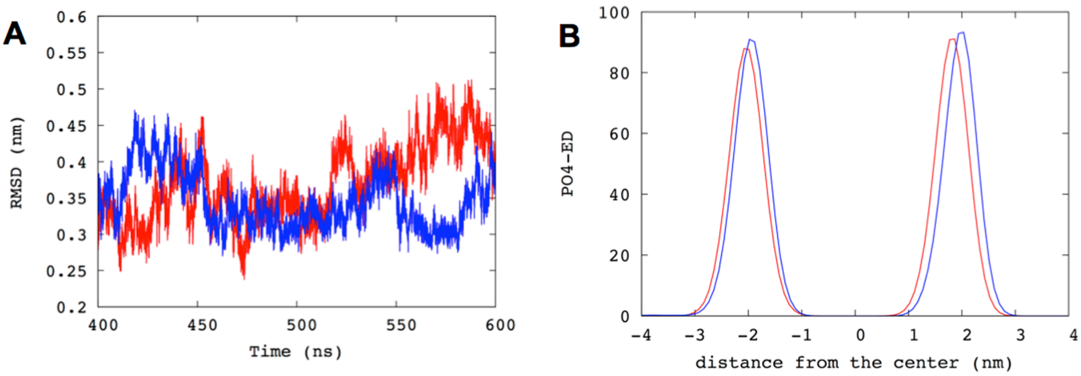

Supporting Information S12 Fig. Changing the ratio between the lipids in system II. The POPC:POPE ratio in **II** is 1 : 1. We consider here a system (**II$_{1.2}$**) that is the same as **II,** except that the ratio of POPC:POPE is 1 : 1.2. For this system, we carry out 600 ns MD simulations. **A)** Cα root-mean-square-deviations (RMSDs) of the receptor's Cα atoms of **II** (red) and **II$_{1.2}$** (blue). **B)** MD-averaged density distribution of the phosphate groups (PO4-ED) in the two systems calculated over the 400ns-600ns interval. The color coding is the same as in **A)**. The peak-to-peak distance, a measure of the bilayer thickness, is 4.0 and 3.9 for **II$_{1.2}$** and **II**, respectively**.** The RMSD matrices and the MD structures of the receptors in **II$_{1.2}$**, as collected in the 400ns-600ns interval, are very similar to those of **II** in the same interval (data not shown).
