# Supplementary material for: Binding of the Antagonist Caffeine to the Human Adenosine Receptor hA2AR in Nearly Physiological Conditions
Source: PLoS One. 2015 May 20;10(5):e0126833. doi: 10.1371/journal.pone.0126833 (PMC4439127; doi:10.1371/journal.pone.0126833)
Supplement: S1 Table — (PDF) [file pone.0126833.s013.pdf]

Supporting Information S1 Table. Available X-ray structures of $hA_{2A}R$. X-ray resolution, mutations and missing regions are reported. Underlined structures are the templates used for constructing the model corresponding to the 1-329 residues of $hA_{2A}R$ target sequence. For the 4 used templates, bound ligand and all-atom RMSD of the binding cavity with respect 3RFM are also shown. 20 residues within 6.5 Å of CFF in 3RFM were considered for calculation of binding cavity RMSD.

PDB ID	X-ray resolution [Å]	Organism	Mutations	Sequence length	Missing residues	Bound ligand	binding cavity RMSD [Å]
2YDO[1]	3.00	Homo sapiens	L48A; A54L; T65A; Q89A; N154A	1-325	1-5; 214-223; 325		
3REY[2]	3.31	Homo sapiens	A54L; T88A; R107A; K122A; L202A; L235A; V239A; S277A	1-329	1-6; 150-157; 306-329		
3PWH[2]	3.30	Homo sapiens	A54L; T88A; K122A; V239A; R107A; L202A; L235A; S277A	1-329	1-6; 150-157; 306-329		
<u>2YDV[1]</u>	2.60	Homo sapiens	L48A; A54L; T65A Q89A; N154A	1-325	1-2; 215-222	NEC agonist	1.448
3VGA[3]	3.10	Homo sapiens	N154Q	1-326	1-5; 149-155; 309-326		
<u>3VG9[3]</u>	2.70	Homo sapiens	N154G	1-326	1-5; 149-155; 310-326	zm241385 antagonist	1.328
<u>3EML[4]</u>	2.60	Homo sapiens	No mutation	1-488	-14-2; 149-155; 311-326	zm241385 antagonist	1.113
3UZC[5]	3.34	Homo sapiens	A54L; T88A; R107A; K122A; L202A; L235A; V239A; S277A	1-329	1-6; 150-157; 306-329		
3UZA[5]	3.27	Homo sapiens	A54L; T88A; R107A; K122A; L202A; L235A; V239A; S277A	1-329	1-6; 150-157; 306-329		
3QAK[6]	2.71	Homo sapiens; <u>Expressed in</u> Enterobacteria phageT4	C54T; C97A	1-488	-14-2; 149-157; 309-326		
<u>4EIY[7]</u>	1.80	Homo sapiens; <u>Expressed in</u> Escherichia coli	M29W; H124I; R128L	1-447	-24--2; 308-326	zm241385 antagonist	1.139
<u>3RFM</u>[2]	3.60	Homo sapiens	A54L; T88A; R107A; K122A L202A; L235A; V239A; S277A	1-329	1-6; 150-157; 306-329	CFF antagonist	

Supporting References

1. Lebon G, Warne T, Edwards PC, Bennett K, Langmead CJ, Leslie AGW, et al. (2011) Agonist-bound adenosine A2A receptor structures reveal common features of GPCR activation. Nature 474: 521-525.
2. Doré Andrew S, Robertson N, Errey James C, Ng I, Hollenstein K, Tehan B, et al. (2011) Structure of the adenosine A2A receptor in complex with ZM241385 and the xanthines XAC and caffeine. Structure 19: 1283-1293.
3. Hino T, Arakawa T, Iwanari H, Yurugi-Kobayashi T, Ikeda-Suno C, Nakada-Nakura Y, et al. (2012) G-protein-coupled receptor inactivation by an allosteric inverse-agonist antibody. Nature 482: 237-240.
4. Jaakola V-P, Griffith MT, Hanson MA, Cherezov V, Chien EYT, Lane JR, et al. (2008) The 2.6 Angstrom crystal structure of a human A2A adenosine receptor bound to an antagonist. Science 322: 1211-1217.
5. Congreve M, Andrews SP, Doré AS, Hollenstein K, Hurrell E, Langmead CJ, et al. (2012) Discovery of 1,2,4-triazine derivatives as adenosine A2A antagonists using structure based drug design. Journal of Medicinal Chemistry 55: 1898-1903.
6. Xu F, Wu H, Katritch V, Han GW, Jacobson KA, Gao Z-G, et al. (2011) Structure of an agonist-bound human A2A adenosine receptor. Science 332: 322-327.
7. Liu W, Chun E, Thompson AA, Chubukov P, Xu F, Katritch V, et al. (2012) Structural basis for allosteric regulation of GPCRs by sodium ions. Science 337: 232-236.
