# Supplementary material for: Binding of the Antagonist Caffeine to the Human Adenosine Receptor hA2AR in Nearly Physiological Conditions
Source: PLoS One. 2015 May 20;10(5):e0126833. doi: 10.1371/journal.pone.0126833 (PMC4439127; doi:10.1371/journal.pone.0126833)
Supplement: S2 Table — (PDF) [file pone.0126833.s014.pdf]

Supporting Information S2 Table. Selected membrane properties of systems I-III. For each system, the receptor-to-lipid hydrogen bonding occupancy ($\rho_{\mathrm{HB}}^{\mathrm{prt-lip}}$), the lipid-to-lipid hydrogen bonding occupancy ($\rho_{\mathrm{HB}}^{\mathrm{lip-lip}}$), the lipid-to-water hydrogen bonding occupancy ($\rho_{\mathrm{HB}}^{\mathrm{lip-sol}}$), and the average area per lipid (APL) are averaged over the last 400 ns of MD simulated time.

**Index**	**I**	**II**	**III**
$\boldsymbol{\rho_{\mathrm{HB}}^{\mathrm{prt-lip}}}$	35/4381	35/4389	37/4037
$\boldsymbol{\rho_{\mathrm{HB}}^{\mathrm{lip-lip}}}$	0	317/3968	238/3616
$\boldsymbol{\rho_{\mathrm{HB}}^{\mathrm{lip-SOL}}}$	3045/30799	3084/31212	2866/29319
**APL (nm^2)**	0.61	0.56	0.50
