# Supplementary material for: Binding of the Antagonist Caffeine to the Human Adenosine Receptor hA2AR in Nearly Physiological Conditions
Source: PLoS One. 2015 May 20;10(5):e0126833. doi: 10.1371/journal.pone.0126833 (PMC4439127; doi:10.1371/journal.pone.0126833)
Supplement: S1 Text — (PDF) [file pone.0126833.s015.pdf]

Supporting Information S1 Text. Description of most populated CFF binding poses A-D.

The A pose (CFF orientational flipping angle ~80-140 degrees) is characterized by the flipping of CFF orientation to the right side relatively to the pose in the X-ray structure. O1@CFF forms hydrogen bond with ND2@N253 and hydrophobic interaction with residues A63, V84, F168, Q169, M177, L249, M270, and I274.

The B pose (CFF orientational flipping angle ~180 degree) is characterized by the CFF bicyclic core inversion with respect to the pose in the X-ray structure. The CFF core forms π-stacking with F168 and hydrophobic interaction with A63, I66, S67, V84, L85, F168, Q169, M177, and H264. Finally O2@CFF forms one water-mediated hydrogen bond with ND2@N253.

The pose C (CFF orientational flipping angle ~120-160 degree) is characterized by the flipping of CFF orientation to the left side relative to the pose in the X-ray structure. The CFF core forms π-stacking interaction with F168. The hydrogen bonding interaction between O2@CFF and NE2@H278 is mediated by one replaceable water molecule. S67, V84, T88, F168, M177, N181, L249, H250, N253, M270, and I274 form hydrophobic interaction with CFF.

The D pose (CFF orientational flipping angle ~0 degree) resembles the pose of CFF in the X-ray structure [1] (RMSD = 0.8 Å). O2@CFF forms a H-bond with ND2@N253. O1@CFF forms a water-mediated H-bond with NE2@H278. I66, V84, F168, Q169, M177, W246, L249, M270, and I274 form hydrophobic interaction with CFF.

Supporting References

1. Doré Andrew S, Robertson N, Errey James C, Ng I, Hollenstein K, Tehan B, et al. (2011) Structure of the adenosine A2A receptor in complex with ZM241385 and the xanthines XAC and caffeine. Structure 19: 1283-1293.
