# Supplementary material for: Binding of the Antagonist Caffeine to the Human Adenosine Receptor hA2AR in Nearly Physiological Conditions
Source: PLoS One. 2015 May 20;10(5):e0126833. doi: 10.1371/journal.pone.0126833 (PMC4439127; doi:10.1371/journal.pone.0126833)
Supplement: S2 Text — (PDF) [file pone.0126833.s016.pdf]

Supporting Information S2 Text. Lateral diffusion coefficient of cholesterol molecules.

To calculate diffusion coefficients from MD simulations, one should use the NVE ensemble. Unfortunately MD simulations based on Gromacs package [1], used here for all calculations, encounter difficulties in reproducing such ensemble. However, a previous study showed that the calculated diffusion coefficients in the NVT ensemble are comparable to that calculated in the NVE one, if one uses the Nosé-Hoover thermostat [2] with relatively small relaxation times. Based on these findings, and keeping in mind that the calculations here are meant for relative comparisons and not for predictions of absolute values, we calculated the lateral diffusion coefficient of system **III**, by carrying out 50 ns in the NVT ensemble. The computational set-up described is the same of the one described in the Method section a part from the removal of the Andersen-Parrinello-Rahman Barostat [3]. Therefore the relaxation time used is 0.5 ns (see methods). The lateral mean square displacement (MSD) of the cholesterol molecules is calculated using the Einstein relation [4], which is then used to determine the lateral diffusion coefficient by linear regression.

Supporting References

1. Hess B, Kutzner C, Van Der Spoel D, Lindahl E (2008) GROMACS 4: algorithms for highly efficient, load-balanced, and scalable molecular simulation. Journal of Chemical Theory and Computation 4: 435-447.
2. Hünenberger P (2005) Thermostat Algorithms for Molecular Dynamics Simulations. Advanced Computer Simulation: Springer Berlin Heidelberg. pp. 105-149.
3. Parrinello M, Rahman A (1981) Polymorphic transitions in single crystals: A new molecular dynamics method. Journal of Applied Physics 52: 7182-7190.
4. Allen MP, Tildesley DJ, Banavar JR (2008) Computer simulation of liquids. Physics Today 42: 105-106.
